# Structural Disorder in High-Spin {Co^II^_9_W^V^_6_} (*Core*)-[Pyridine N-Oxides] (*Shell*) Architectures

**DOI:** 10.3390/molecules25020251

**Published:** 2020-01-08

**Authors:** Michal Liberka, Jedrzej Kobylarczyk, Robert Podgajny

**Affiliations:** Faculty of Chemistry, Jagiellonian University in Kraków, Gronostajowa 2, 30-387 Kraków, Poland; michal.liberka@gmail.com (M.L.); jedrzej.kobylarczyk@uj.edu.pl (J.K.)

**Keywords:** magnetic coordination materials, crystal engineering, polynuclear clusters, surface decoration, molecular disorder, octacyanidometalates

## Abstract

The combinations of Co(II), octacyanidotungstate(V), and monodentate pyridine *N*-oxide (pyNO) or 4-phenylpyridine *N*-oxide (4-phpyNO) led to crystallization of novel crystalline phases {Co^II^[Co^II^_8_(pyNO)_12_(MeOH)_12_][W^V^(CN)_8_]_6_} (**1**) and {Co^II^[Co^II^_8_(4-phpyNO)_7_(MeOH)_17_][W^V^(CN)_8_]_6_}·7MeOH·(4-phpyNO)_3_ (**2**). In both architectures, metal–cyanide clusters are coordinated by *N*-oxide ligands in a simple monodentate manner to give the spherical objects of over 1 nm core diameter and about 2.2 nm (**1**) and 3 nm (**2**) of the total diameter, terminated with the aromatic rings. The supramolecular architecture is dominated by dense and rich π–π interaction systems. Both structures are characterized by a significant structural disorder in ligand shell, described with the suitable probability models. For **1**, the π–π interactions between the pyNO ligands attached to the same metal centers are suggested for the first time. In **2**, 4-phpyNO acts as monodentate ligand and as the crystallization molecule. Magnetic studies indicate the high-spin ground state due to the ferromagnetic interactions Co(II)–W(V) through the cyanido bridges. Due to the high symmetry of the clusters, no signature of slow magnetic relaxation was observed. The characterization is completed by solid-state IR and UV–Vis–NIR spectroscopy. The conditions for the stable M_9_M’_6_-based crystals formation are synthetically discussed in terms of the type of capping ligands: monodentate, bridging, and chelating. The potential of the related polynuclear forms toward the magnetism-based functional properties is critically indicated.

## 1. Introduction

Cyanido-bridged 0D systems of the various linear, polygon, or cluster topologies and 1D chains or ribbons were recently presented in searching for molecular platforms suitable for single-molecule magnet functions. The critical underlying prerequisite—axial magnetic anisotropy—has been shaped through cyanide-bridging of suitable d and f metal ion and dedicated coordination surrounding. For example, among the frequently exploited building blocks, one can indicate 6-coordinated *fac*-[M(L)(CN)_3_]^−^ (M—Fe^III/II^, Co^III/II^; l-hydrotris(pyrazol-1-yl)borate, tetra(pyrazol-1-yl)borate) moieties with trigonal distortion [1,2,3,4], octahedral Co^II^ with axial deformation [5,6], *trans*-[M(L)(CN)_2_] (M–Fe, Cr; l-planar pentadentate ligands) heptagonal bipyramidal moieties [7,8] or intrinsically anisotropic 4d or 5d heavy metal precursors [9], and lanthanide complexes [10,11].

Along this line, investigation of the crystalline phases composed of six capped body-centered cube Co_9_M’_6_ clusters as molecular platforms for magnetic relaxations suggests the following image of structure–properties correlations. Twelve compounds were studied structurally and magnetically, counting the original {Co_9_M_6_(MeOH)_24_}∙solv (M = Mo, W) phases [12] and those equipped with various ligands: chelating, 2,2′-bipyridine-*N*-oxide (2,2′-bpmo) [13], 2,2′-bipyridine-*N*,*N*’-dioxide (2,2′-bpdo) [14], and methylpyridinemethanol (mpm) [15], as well as bridging 4,4′-bipyridine-*N*-oxide (4,4′-bpmo) [16], 4,4′-bipyridine-*N*,*N*’-dioxide (4,4′-bpdo) [17], and pyrazine-*N*-oxide (pzmo) [16]. The unequivocal direct fitting of ln*τ*(*T*^−1^) point set using the Arrhenius law was possible only in the case {Co_9_W_6_(2,2′-bpdo)_6_(MeOH)_12_}{Co_9_W_6_(2,2′-bpdo)_7_(MeOH)_10_}∙8H_2_O∙2MeCN∙27MeOH to give the best ∆*E*/*k*_B_ of 30.0(8) K along the whole series [14]. Interestingly, the Cole–Cole plot indicated that only half of the clusters took part in the relaxation process. The relaxation behavior was then assigned solely to the Co_9_W_6_(2,2′-bpdo)_7_(MeOH)_10_ cluster with only one external Co site free of chelating ligand, and related to the sufficient axial character of the cluster core. No correlation was found between the magnetic ac data and the intercluster distance, quality of intercluster contacts, or composition of the first coordination sphere.

The inherent issue along the above series was the tuning of the extent of cluster coverage, size, shape, and quality of the peripheral regions resulting from the molecular structure and coordination modes of external ligands. The bis-chelating diimine-*N*-oxide and other related ligands increase the hydrophobic character of the external regions and set the average diameter between 1.9 and 2.2 nm, compared to the diameter of 1.6 nm for non-chelated {Co_9_W_6_(MeOH)_24_}. On the contrary, ditopic 4,4′-bridging congeners provides the star-like branched coordination backbones (discrete or chain-like) with molecular diameters between ca. 2.1 and 2.7 nm with the remote hydrophilic functions exposed at the peripheral parts. Such forms tend to create polymeric forms; however, they can be also expected to bind the external remote functional coordination units. In general, the variety of multifaceted intercluster supramolecular synthons were observed in the related crystal structures, and rather without the straightforward correlation between the ligand type and related intercluster separation.

Having in mind the above experiences with the Co_9_W_6_ cluster family, we performed the decoration of the Co_9_W_6_ core with the monodentate pyridine *N*-oxide (pyNO) or 4-phenylpyridine *N*-oxide (4-phpyNO), which provides the singly and presumably less tightly bonded hydrophobic ends of pyridine and phenyl group. Driven by curiosity, we aimed to test the new type of ligands for alternative coordination modes and supramolecular organization toward (rather a serendipitous) occurrence of a higher degree of magneto-structural asymmetry that allows unblocking the slow magnetic relaxations. We present the crystal structures, spectroscopic characterization, and magnetic properties of new {Co^II^[Co^II^_8_(pyNO)_12_(MeOH)_12_][W^V^(CN)_8_]_6_} (1) and {Co^II^[Co^II^_8_(4-phpyNO)_7_(MeOH)_17_][W^V^(CN)_8_]_6_}·7MeOH·(4-phpyNO)_3_ (2) congener.

## 2. Results and Discussion

### 2.1. Structural Studies

**Figure 1:**
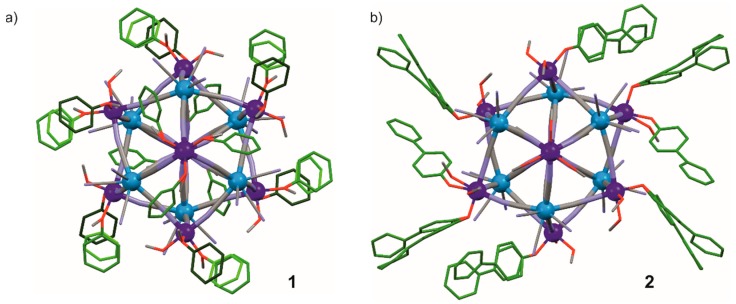
Pentadecanuclear clusters in the crystal structure of 1 (**a**) and 2 (**b**). Hydrogens atom, crystallization solvents, and non-coordinated ligands were omitted for clarity. Legend: Co—navy blue; W—light blue; C—dark gray; N—violet; O—red; C and N in pyNO and 4-phpyNO ligands-dark green (75% occupancy), green (50% occupancy), and light green (25% occupancy) (for details see Supplementary Materials).

Both new compounds **1** and **2** were obtained in crystalline form. All samples were characterized by IR and UV–Vis spectra, TGA, and CHN elemental analysis (Figures S1–S3, ESI†) followed by a single-crystal X-ray diffraction analysis (Figure 1, Table 1, and Figures S4–S8, Tables S1 and S2, ESI†). The symmetrically independent parts are presented in Figures S4 and S5 (ESI†). The results of data collection and structure refinement have been summarized in Table 1. Compounds 1 and 2 crystallizes in the trigonal R$\bar{3}$ and triclinic P$\bar{1}$ crystallographic space group, respectively. The crystal structures of both compounds consist of pentadecanuclear cyanide-bridged clusters {Co_9_W_6_L_x_(MeOH)_24-x_} (Figure 1) due to the cluster surface decoration with monodentate pyridine *N*-oxide (pyNO, L^1^, x = 12) for **1** or 4-phenylpyridine *N*-oxide (4-phpyNO, L^2^, x = 7) for **2**. The cluster core in both cases is analogous to the six capped body-centered cubes of broad M_9_M’_6_ family. In both cases, the central [Co1(μ-NC)_6_]^2+^ moiety with almost perfect octahedron geometry (Tables S3 and S4, ESI†) is surrounded by six cyanide-bridges directed toward six [W(µ-CN)_5_(CN)_3_]^3*−*^ blocks located in the vertices of super-octahedron. The eight remaining Co^2+^ ions are located in the corners of the super-cube, each accepting three cyanide-bridges from the neighboring W centers, reaching the general composition fac-[Co^II^(µ-NC)_3_L_y_(MeOH)_3-y_]^−^. In compound **1**, two crystallographically independent external fac-[Co2/3(μ-NC)_3_(pyNO)_1.5_(MeOH)_1.5_]^2+^ moieties are distinguished, with occupancy 0.25, 0.5, 0.75, or 1.0 of the organic and solvent ligands (see Figure S4, ESI†). In compound **2**, four crystallographically independent external cobalt moieties were found: one fac-[Co3(μ-NC)_3_(MeOH)_3_]^2+^, two fac-[Co2/5(μ-NC)_3_(4-phpyNO)(MeOH)_2_]^2+^, and one fac-[Co4(μ-NC)_3_(4-phpyNO)_1.5_(MeOH)_1.5_]^2+^ (with occupancy 0.5 or 1.0 of the organic and solvent ligands, see Figure S5, ESI†). All Co^2+^ ions exhibit practically O_h_ geometry (Tables S3 and S4, ESI†). The Co-N/O distances in **1** and **2** are in the range of 2.061*–*2.123 Å, which is typical for Co_9_W_6_-based clusters with monodentate ligands. All [W(µ-(CN)_5_(CN)_3_]^3−^ ions connect five cobalt ions, while three cyanide ligands are terminal. Chosen distances and angles are collected in Tables S1 and S2 (ESI†). All values are in line with the related literature data [13,14,15,16,17].

The application of a new type of ligands to fifteen-centered clusters allowed obtaining a completely new character of the ligand shell. In **1**, pyNO ligands connected to the Co3 moieties interacts with each other with π–π interactions, which has not been seen in M_9_M’_6_L_x_ clusters with monodentate coordination of ambidentate linkers (L = pyrazine mono-*N*-oxide (pzmo)) and 4,4-bipyridine mono-*N*-oxide (4,4’-bpdo, Figure S6, ESI†) [16]. Supramolecular arrangement is also dominated by this type of interaction, but there are also weak O3_MeOH_∙∙∙N8_CN_ hydrogen bonds in the direction [001]. Each cluster is surrounded symmetrically by six other clusters, creating a densely packed 3D supramolecular architecture. The shortest intercluster distance is 5.22 Å, which conforms to the absence of additional solvent molecules in the molecular architecture. No interactions between 4-phpyNO ligands coordinated to the same metallic center are observed in the case of **2**, which is in line with a relatively large freedom of rotation of the phenyl ring. The crystal structure of **2** is completed with additional crystallization MeOH molecules, creating a network of hydrogen bond synthons, as well as uncoordinated 4-phpyNO molecules (with occupancy 0.5 or 1.0; Figure S6, ESI†). The face-to-face or edge-to-face π–π interactions between coordinated and uncoordinated ligands form supramolecular layers perpendicular to the [010] direction, which are additionally stabilized by O5M_MeOH_–N38_CN_ hydrogen bonds. Longer ligand and also the presence of uncoordinated 4-phpyNO molecules in **2** compared to **1** result in the larger separation of clusters. The shortest distance between them is 7.23 Å, and the clusters are not distributed in such a symmetrical manner as in the case of **1**. The structural uniformity has been confirmed using powder X-ray diffraction data (Figure S9, ESI†).

To discuss the above structural description, we consider below the observations for the entire M_9_M’_6_L_x_ family of compounds, focusing on (i) the molar ratios in the parent solution and in the final product, and (ii) on the structural disorder in the ligand shell. All previous reports on functionalized fifteen-centered cluster cores were associated with *O*,*O*-, *N*,*O*-, *N*,*N*-, or *N*,*N*,*N*-donor ligands providing their monodentate (**m**), bidentate (**b**), or tridentate (**t**) *local* coordination: Mn_9_W_6_L_x_ (L = 4,4′-bipyridine, x = 4, **m**; *trans*-1,2-di(4-pyridyl)ethylene, x = 5, **m**; 4,4′-dipyridyl disulfide, x = 4, **m**; 4,4′-di-tert-butyl-2,2′-bipyridine, x = 8, **b;** 4,7-di-phenyl-1,10-phenantroline, x = 8, (**b**), Fe_9_M′_6_(Me_3_tacn)_8_ (Me_3_tacn = 1,4,7-trimethyl-1,4,7-triazacyclononane; M′ = W, Re, **t**), Co_9_W_6_L_x_ (L = 4,4′-bipyridine di-*N*-oxide, x = 12, **m**; (R/S)methylpyridinemethanol, x = 8, **b**; 2,2′-bipyridine di-*N*,*N*-oxide, x = 6/7, **b**; 2,2′-bipyridine mono-*N*-oxide, x = 6 or 8 **b**; 4,4′-bipyridine mono-*N*-oxide, x = 4 or 6, **m**; pyrazine mono-*N*-oxide, x = 5, **m**), Ni_9_W_6_L_x_ (L = 2,2′-bipyridine, x = 8, **b**; 4,4′-dimetyl-2,2′-bipyridine, x = 8, **b**; 5,5′-dimetyl-2,2′-bipyridine, x = 8, **b**; 4,4′-di-tert-butyl-2,2′-bipyridine, x = 8, **b**; 3,4,7,8-tetrametyl-1,10-phenantroline, x = 6, **b**; 2,2′-bi(4,5-dihydrothiiazine), x = 8, **b**; (*R/S*)-2-(1-hydroxyethyl)pyridine, x = 8, **b**), Ni_9_Mo_6_L_x_ (L = 2,2′-bipyridine, x = 8, **b**; 3,4,7,8-tetrametyl-1,10-phenantroline, x = 6, **b**), and Co_1_Cu_8_W_6_(Me_3_tacn)_8_, **t** [12,13,14,15,16,17,18,19,20]. The formation of stable crystalline product depended strongly on L: M^2+^ molar ratio in solution. For the chelating ligands, the L:M^2+^ of 1:1 was sufficient to observe a complete or an almost complete capping of the peripheric M^2+^ sites, with the resulting L:M^2+^ ratio of 8:8 (prevalently), 7:8 or 6:8 in the cluster coverage (in the case of bis-chelating ligands), and 8:8 (in the case of tridentate Me_3_tacn), and 16:8, 14:8, or 12:8, and 24:8, respectively, counting separately each coordinated donor atom. For the linker-type ligands, the effective growth of the crystals was observed only by some excess of L, 1.5:1, 5:1, 7:1, or 20:1, with respect to the M^2+^, to give the cluster coverage ratio L:M^2+^ between 12:8 and 4:8. In this work, for the first time, we have the opportunity to present compounds based on 15-centered clusters containing exclusively monodentate (without the bridging function) ligands, pyNO, and 4-phpyNO. To achieve effective crystallization, the use of a minimum of 30-fold excess of the ligand was required, with the resulting coverage L: M^2+^ ratio 12:8 (**1**) and 7:8 (10:7 ratio involving all 4-phpyNO in the crystal structure) (**2**) observed. Although no direct evidence was shown for the presence of complete 15-nuclear species in solution, the thermodynamic equilibria can be inferred to operate in solution, 15-nuclear cores acting as super-complexes with eight triple coordination sites located at the corners of super-cube sublattice. The above diversification in the ease of the crystal formation is in agreement with the thermodynamic prediction of complex stability considering the chelate effect, or “anchoring” due to the bridging, against the “simple” coordination of monodentate ligand. The ligand shell disorder in **1** and **2** is unprecedented along the entire family, and may be understood in terms of the degree of M–L bond rigidity and intercluster interactions inscribed in the ligand structure and M–L bonding mode. The longer linker ligands (e.g., 4,4′-bipyridine mono-*N*-oxide, 4,4′-bpmo; 4,4′-bipyridine di-*N*-oxide, 4,4′-bpdo) [16,17] form quite easily the bridging connections between clusters, and/or participate in the hydrogen bond supramolecular interaction network. On the other hand, the convergent bidentate ligand (frequently equipped with the remote substituents, e.g., 4,4′-dimetyl-2,2′-bipyridine, 4,4′-di-tert-butyl-2,2′-bipyridine, or 4,7-di-phenyl-1,10-phenantroline) [20] fill the intercluster space with the π–π synthons or van der Waals contacts. In both cases, the degree of freedom is strongly limited, which prevents the disorder. Interestingly, the dataset for pyrazine mono-*N*-oxide (pzmo) ligand [16], the shorter analog of 4,4′-bpmo, fairly resembles that of pyNO in **1**: (i) one of the pzmo ligands reveals severe positional disorder, and (ii) preparation of the product requires the ratio L:Co^2+^ ratio of 20:1, a little less than 30:1 in **1**. The occurrence of disorder in both cases may be correlated with the small volumes of both ligands, whereas the differences are definitely related to the natural preferences for intermolecular interactions. Coming to the compound **2**, the monodentate or crystallization form of 4-phpyNO moiety can be confronted with the diversity of coordination modes, bridging and monodentate, noted also both for Co_9_W_6_(4,4′-bpmo)_4;6_ and Co_9_W_6_(4,4′-bpdo)_12_.

### 2.2. Magnetic Properties

**Figure 2:**
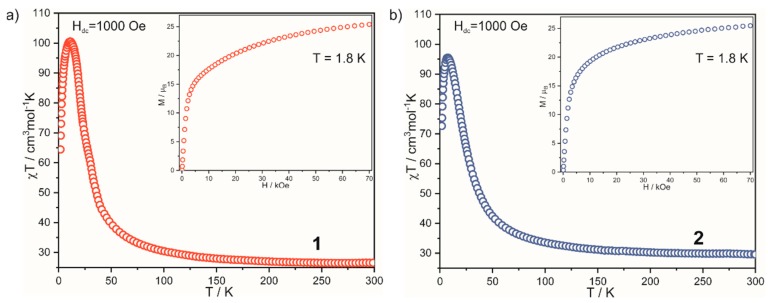
Temperature dependence of *χT* for 1 (**a**) and 2 (**b**) measured at H = 1000 Oe. Inset: isothermal magnetization at *T* = 1.8 K for both compounds, 1 (**a**) and 2 (**b**).

Temperature dependences of the molar magnetic susceptibility χT(T) in the T = 1.8–300 K and H_dc_ = 1 kOe for **1** and **2** are presented in Figure 2. The room-temperature χT value for cyanide-bridged cluster of **1** and **2** is 26.57 and 29.58 cm^3^ mol^−1^ K, respectively, which are in the range 26.25–33.00 expected for combined contribution of nine ^HS^Co^II^ (with S = 3/2 and g = 2.4–2.7) and six W^V^ ions (with S = 1/2 and g = 2.0). The continuous increase of χT value to 100.50 and 95.49 cm^3^∙mol^-1^∙K at 10.25 and 7.58 K for **1** and **2**, respectively, indicates ferromagnetic superexchange interactions between ion centers connected by CN-bridges. After reaching the maximum value, the signal drops to 64.47 and 72.63 cm^3^∙mol^−1^∙K at 1.8 K for **1** and **2**, respectively, which is related to the intercluster interaction and zero-field splitting effect on ^HS^Co^II^ ions. The maxima of χT correspond well to the theoretical value of 92.12 cm^3^mol^-1^K expected for isolated spins S_av_ = 15/2 with g_av_ = 3.40, when calculations with standard parameters for W^V^ (S = 1/2, g = 2.0) and effective spin approach for ^LT^Co^II^ (S = 1/2, g = 13/3) are used. The insets in Figure 2 show isothermal field dependences of magnetization M(H) in the magnetic field range H_dc_ = 0–70 kOe at 1.8 K to support the ferromagnetic type of interaction. Almost complete saturation is achieved with the values of 25.40 (**1**) and 25.47 (**2**) N_β_ at 70 kOe, which corresponds to the expected value of 25.5 μ_B_ calculated for S = 15/2 with g = 3.4. The above characteristics and related numbers are in line with previous observations along the family [13,14,15,16,17].

Unfortunately, no signature of slow magnetic relaxation was observed in zero or non-zero H_dc_ field and this can be related to the high symmetry of the coordination backbone (Figure S10, ESI†). Along with the twelve compounds shown previously, we have indicated that distinct characteristics with the χ” (T) maxima above T = 1.8 K could be observed only in the case of covering with 7 2,2′-bpdo or 24 MeOH. We explained their presence with the suitable magnetic anisotropy due to asymmetry of coverage and/or to significant deformation of the central Co moieties [16] described by SHAPE analysis. Considering **1** and **2**, the attempt of serendipitous induction of coverage asymmetry failed. Coming to the SHAPE analysis, rather highly symmetric central [Co (µ-NC)_6_] moieties was indicated in both compounds, either (Figure S11, Tables S6 and S7, ESI†). Some diversification was observed for the external Co units; however, this was not followed by the SMM behavior. Thus, the complete information set for the diverse fourteen compounds in this matter leads us to the conclusion that Co_9_W_6_ is rather difficult to functionalize toward outstanding SMM behavior, either by the ligand decoration or by the plausible accidental asymmetric truncation/extension of the coordination skeleton.

## 3. Experimental

### 3.1. Reagents and Materials

K_4_[W(CN)_8_]⋅2H_2_O and TBA_3_[W^V^(CN)_8_] were obtained according to the standard procedure. Firstly, K_4_[W^IV^(CN)_8_]∙2H_2_O was obtained from K_2_WO_4_ via the combined reduction–cyanation–oxidation protocol involving NaBH_4_, perhydrol, and KCN in H_2_O/CH_3_COOH media [21]. Then, K_3_[W^V^(CN)_8_] was obtained through the oxidation of K_4_[W^IV^(CN)_8_] in aqueous-acidic media (HNO_3_), followed by immediate precipitation of Ag_3_[W^V^(CN)_8_] using AgNO_3_. Na_3_[W^V^(CN)_8_]∙4H_2_O was crystallized from the solution acquired after the solid-state solution metathesis between NaCl and Ag_3_[W^V^(CN)_8_]_(s)_ in H_2_O. Finally, TBA_3_[W^V^(CN)_8_] was precipitated from the aqueous solution of Na_3_[W^V^(CN)_8_] using TBACl. Inorganic substances were purchased from commercial sources (Sigma Aldrich, Poznań, Poland, Alfa Aesar, Kandel, Germany, Acros Organics, Poznań, Poland). Ligands pyridine *N*-oxide (pyNO, 95%) and 4-phenylpyridine *N*-oxide (4-phpyNO, 98%) were purchased from Sigma Aldrich and were used without further purification.

### 3.2. X-ray Diffraction Analysis

Single-crystal X-ray diffraction experiment of **1** and **2** was performed using Bruker D8 Quest Eco diffractometer equipped with Photon50 CMOS detector with standard Mo (Kα, λ = 0.71073 Å) radiation source, graphite monochromator, and Oxford Cryosystem cooling system. Measurements were performed at 100 K for crystals of **1** and **2**, covered by NVH immersion oil in order to prevent the exchange of crystallization solvent and structure degradation. The structures **1** and **2** were solved using SHELXT and refined with full-matrix least-squares procedure on F^2^ using SHELXL with Olex2 graphic interface. All non-hydrogen atoms were refined with anisotropic parameters. Positions of hydrogen atoms were assigned at the idealized positions using the riding model. Due to a large structural disorder on pyNO ligands in **1** and 4-phpyNO ligands in **2**, some restraints (DFIX, DANG, SIMU, DELU) on carbon or nitrogen atoms were applied. Relatively high residual density in the vicinity of some of the tungsten atoms is probably caused by an unaccounted disorder of cyanide groups. The results of data collection and structure refinement of **1** and **2** have been summarized in Table 1. CCDC reference numbers for the crystal structure are 1972361 (**1**) and 1972362 (**2**). Structural figures were prepared using the Mercury software. Powder X-ray diffraction data for **1** and **2** were collected using a Bruker D8 Advance Eco powder diffractometer equipped with Cu (Kα, λ = 1.5419 Å) radiation source. The measurements were conducted for polycrystalline samples in the mother solution inserted into a glass capillary (0.5 mm) [22,23].

### 3.3. Physical Techniques and Calculations

Elemental analyses of CHNS were performed on an Elemental Vario Micro Cube CHNS analyzer. The infrared absorption spectra were collected on the selected single crystals on an FT-IR Thermo Scientific Nicolet iN10 microscope. UV–Vis absorption spectra were collected on thin films of powder samples dispersed in NVH immersion oil using a Perkin-Elmer Lambda-35 spectrophotometer. The thermogravimetric (TGA) curves were collected for the polycrystalline samples using a Rigaku Thermo Plus TG8120 apparatus with aluminum pans as holders. The data were collected in the temperature range 20–375 °C under air atmosphere with a heating rate of 1 °C per minute. Magnetic data were collected using Quantum Design MPMS-3 Evercool magnetometer. The powder samples were measured in the glass tube covered by a small amount of the mother solution. Diamagnetic corrections from the sample, mother solution, and sample holder were introduced [24]. Continuous Shape Measure Analysis for the coordination sphere of each metal complex was performed using a SHAPE software [25].

### 3.4. Synthetic Procedures

***Synthesis of *1**.** The 14.7 mg (0.06 mmol) of CoCl_2_·6H_2_O was dissolved together with 45 mg (0.04 mmol) of TBA_3_[W(CN)_8_] in 4 mL MeOH. The red solution was stirred for ca. 2 min, and the methanolic (2 mL) solution of 171.2 mg (1.8 mmol) of pyNO ligand was added. The deep-red solution was mixed for another 2 min, filtrated off, and tightly closed. After one day, dark red crystals of **1** appeared. The composition of {Co^II^[Co^II^_8_(pyNO)_12_(MeOH)_12_][W^V^(CN)_8_]_6_} was defined by a single-crystal X-ray diffraction analysis. Phase purity was proved by XRD data. Crystals are stable in mother solution; however, solvent molecules are exchanged for water molecules in air. The formula of the hydrated form {Co^II^_9_[W^V^(CN)_8_]_6_(pyNO)_12_·1MeOH·17H_2_O} (**1^hyd^**) was determined by CHN elemental analysis and TGA measurement. Yield for **1^hyd^**: 41% (based on Co). Elemental analysis. Calc. for C_109_H_98_Co_9_N_60_O_30_W_6_: C, 30.01%; H, 2.26%; N, 19.27%. Found: C, 30.23%; H, 2.41%; N, 19.05%. **1^hyd^** was of poor crystallinity, which precluded reliable crystal structure determination. IR spectrum for **1**. Cyanide stretching vibrations v(C≡N) at 2213, 2172, and 2144 cm^−1^ are related to both bridging and terminal cyanides in [W^V^(CN)_8_]^3−^ building blocks. UV–Vis spectrum for **1**. The wide range of absorption bands (UV—750 cm^−1^) can be explained by the sum of ligand field electronic transitions of [W^V^(CN)_8_]^−3^ ions, d–d electronic transitions of ^HS^Co^II^ ion, and metal-to-metal charge transfer (MMCT) electronic transitions.

***Synthesis of *2**.** The 14.7 mg (0.06 mmol) of CoCl_2_·6H_2_O was dissolved together with 45 mg (0.04 mmol) of TBA_3_[W(CN)_8_] in 4 mL MeOH. The red solution was stirred for ca. 2 min, and the methanolic (2 mL) solution of 308.2 mg (1.8 mmol) of 4-phpyNO ligand was added. The blood-red solution was mixed for another 2 min, filtrated off, and tightly closed. After one day, dark red crystals of **2** appeared. The composition of {Co^II^[Co^II^_8_(4-phpyNO)_7_(MeOH)_17_][W^V^(CN)_8_]_6_}·7MeOH·(4-phpyNO)_3_ was defined by a single-crystal X-ray diffraction analysis. Phase purity was proved by XRD data. Crystals are stable in mother solution; however, solvent molecules are exchanged for water molecules in air. The formula of the hydrated form {Co^II^_9_[W^V^(CN)_8_]_6_(4-phpyNO)_10_·1MeOH·17H_2_O} (**2^hyd^**) was determined by CHN elemental analysis and TGA measurement. Yield for **2^hyd^**: 37% (based on Co). Elemental anal. calc. for C_159_H_128_Co_9_N_58_O_29_W_6_: C, 38.59%; H, 2.61%; N, 16.42%. Found: C, 38.78%; H, 2.85%; N, 16.68%. **2^hyd^** was of poor crystallinity, which precluded reliable crystal structure determination. IR spectrum for **2**. Cyanide stretching vibrations v(C≡N) at 2220, 2174, and 2150 cm^−1^ are related to both bridging and terminal cyanides in [W^V^(CN)_8_]^3−^ building blocks. UV–Vis spectrum for **2**. The wide range of absorption bands (UV—750 cm^−1^) can be explained by the sum of ligand field electronic transitions of [W^V^(CN)_8_]^−3^ ions, d–d electronic transitions of ^HS^Co^II^ ion, and metal-to-metal charge transfer (MMCT) electronic transitions.

***Comment*:** It is necessary to add that only a drastic excess of ligand relative to Co^2+^ ions, here 45:1, caused the equilibrium shift toward the crystallization of the product; a change of the ligand/Co^2+^ ratio results in a reduction of yield and product quality or prevents its formation.

## 4. Conclusions

We have presented two new pentadecanuclear cluster-based compounds: {Co^II^[Co^II^_8_(pyNO)_12_(MeOH)_12_][W^V^(CN)_8_]_6_} (**1**) and {Co^II^[Co^II^_8_(4-phpyNO)_7_(MeOH)_17_][W^V^(CN)_8_]_6_}·7MeOH·(4-phpyNO)_3_ (**2**), obtained in crystalline form through a suitable combination of Co^2+^ and [W(CN)_8_]^3−^ ions and monodentate pyNO or 4-phpyNO ligands. Their crystal structure and magnetic properties were examined. PyNO ligands decorating the cluster core in **1** form densely packed π–π supramolecular structure, in which the symmetrical surrounding of the clusters is observed. In addition, parallel π–π interaction between ligands coordinated to the same ion center is observed, which has not been seen in M_9_M’_6_ clusters with monodentate ligand-based yet. In compound **2**, the 4-phpyNO ligands either decorate the M sites or act as the crystallization molecules. In both structures, a significant ligand shell structural disorder was observed, with the partial occupancy of individual coordinating units (ligands, solvents) of 0.25, 0.50, or 0.75 determined within the solution and refinement model. DC magnetic properties indicate the high-spin ground state due to ferromagnetic Co(II)-W(V) interactions, in agreement with the previous reported Co_9_W_6_ cluster-based compounds. Due to the high symmetrical arrangement of clusters in **1** zero-signal in ac measurements was observed. In **2**, 4-phpyNO longer than pyNO offers more degrees of freedom due to the phenyl ring rotation but it turns out to be insufficient to observe χ’’(T) signal. However, the presented supramolecular building blocks Co_9_W_6_L_x_ can be perceived as a potential source of polynuclear nanometer scale building block for the π–π supramolecular organization of supramolecular networks. The research is underway in the project group.

Going beyond the above Co_9_W_6_-L approach, the magnetic properties can be modified by the topological changes done to six capped body-centered cube topology through the modification of other synthetic conditions. For example, the slow diffusion CoCl_2_, RbCl, and Rb_3_[W(CN)_8_] in water-acetone media leads to the porous-like Co_7_[W(CN)_8_]4Cl_2_·29H_2_O 3D network with 1.4 nm inter-skeletal diameter, containing the tubular wires {Co_5_W_4_} that could be perceived as the in situ-formed 1D secondary building units (SBU) [26]. The long-range magnetic ordering (LRMO) temperature T_C_ = 29 K and coercive field Hc = 5.5 kOe were found. Very similar thick wire 1D arrangement with T_N_ of 3.5 K has been acquired via the unique direct quadruple bridging between Mn_9_W_6_ clusters, assisted by the bridging of dpe linkers [27]. The slow diffusion of water vapor into the mixture of Mn_9_W_6_ and pyrazine-*N*,*N*-dioxide (pzdo) in MeOH leads to the 2D topology closely related to the cluster surface grid, not achieved in the typical aqueous conditions used for the extended [M(CN)_8_]^n−^ networks [28]. The above observations still allow to reasonably consider the acquisition of the large defected discrete Co_x_W_y_^n+/−^ species or single chains based on such motifs, potentially hosting the slow magnetic relaxation properties. Finally, the use of the triazacyclononane (tacn) family allows to afford 15-nuclear and larger related 20-nuclear topologies, which offer the stabilization of paramagnetic [W(CN)_8_]^3−^ anions, [19,29,30] spin-crossover on all nine Fe(II) sites [31], and photomagnetic properties in the case of Cu^II^-[Mo^IV^(CN)_8_]^4−^ species [32].

## Figures and Tables

**Table 1:** Crystal data and structure refinement for **1** and **2**.

Compound	1	2
**Formula**	Co_9_W_6_C_120_H_108_N_60_O_24_	Co_9_W_6_C_183_H_159_N_58_O_34_
**Formula weight/g·mol^−1^**	4408.04	5348.14
**T/K**	100	100
**λ/Å**	0.71073	0.71073
**Crystal system**	Trigonal	Triclinic
**Space group**	R$\bar{3}$	P$\bar{1}$
**Unit cell**		
**a/Å**	28.126(4)	17.179(1)
**b/Å**	28.126(4)	17.513(1)
**c/Å**	18.038(2)	20.029(1)
**α/deg**	90	83.770(1)
**β/deg**	90	79.935(1)
**γ/deg**	120	64.080(1)
***V*/Å^3^**	12,358.0(30)	5333.1(4)
***Z***	3	1
**Calculated density/g·cm^−1^**	1.777	1.665
**Absorption coefficient/cm^−1^**	5.125	3.978
***F(000)***	6381	2622
**Crystal size/mm × mm × mm**	0.41 × 0.27 × 0.18	0.33 × 0.27 × 0.21
***θ* range/deg**	2.48 to 25.38	2.30 to 25.39
**Limiting indices**	−33 < h < 33	−20 < h < 20
	−33 < k < 29	−20 < k < 20
	−21 < l < 21	−23 < l < 23
**Collected reflections**	5210	9900
**Symmetry independent reflections**	4818	18,710
***R_int_***	0.1055	0.0372
**Completeness/%**	99.0	99.3
**Data/restrains/parameters**	4818/463/472	18710/926/1856
**GOF on *F*^2^**	1.093	1.394
**Final *R* indices**	*R[F^2^ > 2σ(F*^2^*)] =* 0.0675	*R[F^2^ > 2σ(F*^2^*)] =* 0.0804
	*wR(F*^2^*) =* 0.1564	*wR(F*^2^*) =* 0.1740
**Largest diff peak and hole/e·Å^3^**	1.851/−1.759	1.409/−2.372

## Supplementary Materials

The following are available online. Figure S1 (IR spectra), Figure S2 (TGA curves), Figure S3 (UV–Vis–NIR absorption spectra), Figures S4 and S5 (Crystal structure illustrations), Figure S6 (Stacking interactions), Figure S7 and Table S8 (Crystal packing), Figure S9 (PXRD patterns), Figure S10 (out-of-phase ac magnetic measurements), Figure S11 (SHAPE maps for Co(II) centers), Table S1 and Table S2 (detailed structural parameters of 3d metal complexes), Table S2 (detailed structural parameters of 3d metal complexes in 2), Table S3 and Table S4 (CSM analysis), Table S5 (H-bonds parameters), Tables S6 and S7 (the overview of the structural parameters).
