# Supplementary material for: Structural Disorder in High-Spin {CoII9WV6} (Core)-[Pyridine N-Oxides] (Shell) Architectures
Source: Molecules. 2020 Jan 8;25(2):251. doi: 10.3390/molecules25020251 (PMC7024233; doi:10.3390/molecules25020251)
Supplement: Supplementary file 1 [file molecules-25-00251-s001.pdf]

Supplementary Information

Article

Structural disorder in high-spin $\{Co_9W_6\}$(*core*)-[pyridine N-oxides](*shell*) architectures

Michal Liberka, Jędrzej Kobylarczyk and Robert Podgajny *

Faculty of Chemistry, Jagiellonian University in Kraków, Gronostajowa 2, 30-387 Kraków, Poland; michal.liberka@uj.edu.pl (M.L.); jedrzej.kobylarczyk@uj.edu.pl (J.K.)

* Correspondence: robert.podgajny@uj.edu.pl; Tel.: +48-12-686-2459 (R.P.)

Received: date; Accepted: date; Published: date

Figure S1. Infrared spectra of **1** and **2** measured at room temperature in the absorption mode for the selected single-crystals in the 4000–700 cm^{-1} range and cyanide stretching vibrations range.

Figure S2. Thermogravimetric curves collected in the temperature range 20 – 375 °C for **1** and **2**. The steps related to the loss of solvent molecules are featured.

Figure S3. UV–Vis–NIR absorption spectra of **1** and **2** measured at room temperature in the 350 - 900 nm range.

Table S1. Detailed structural parameters of 3d metal complexes in **1**.

Table S2. Detailed structural parameters of 3d metal complexes in **2**.

Table S3. Results of Continuous Shape Measure (CSM) analysis for metal complexes in **1**.

Table S4. Results of Continuous Shape Measure (CSM) analysis for metal complexes in **2**.

Figure S4. Crystal structure of **1**.

Figure S5. Crystal structure of **2**.

Figure S6. Stacking interactions in **1** and **2**.

Figure S7. Packing of clusters in the crystal structure of **1**.

Figure S8. View of cluster arrangement in crystal structure of **2**.

Table S5. Hydrogen bonds parameter in **1** and **2**.

Figure S9. Experimental powder X-ray diffraction (PXRD) pattern of **1** and **2** presented in the broad 2Θ range of 3–40° and in the limited low angle 2Θ range of 3–12°.

Figure S10. Frequency dependence of out-of-phase magnetic susceptibility, χ'' at 1.8 K in dc field range 0-2500 Oe for **1** (a) and 0-600 Oe for **2** (b).

Figure S11. (OC-6)-(TPR-6) shape map with the positions of the coordination polyhedral of Co moieties in **1** and **2**.

Table S6. The overview of the structural parameters of central Co moieties in Co_9W_6 clusters in **1** and **2**.

Table S7. The overview of the structural parameters of Co_9W_6 clusters in **1** and **2**.

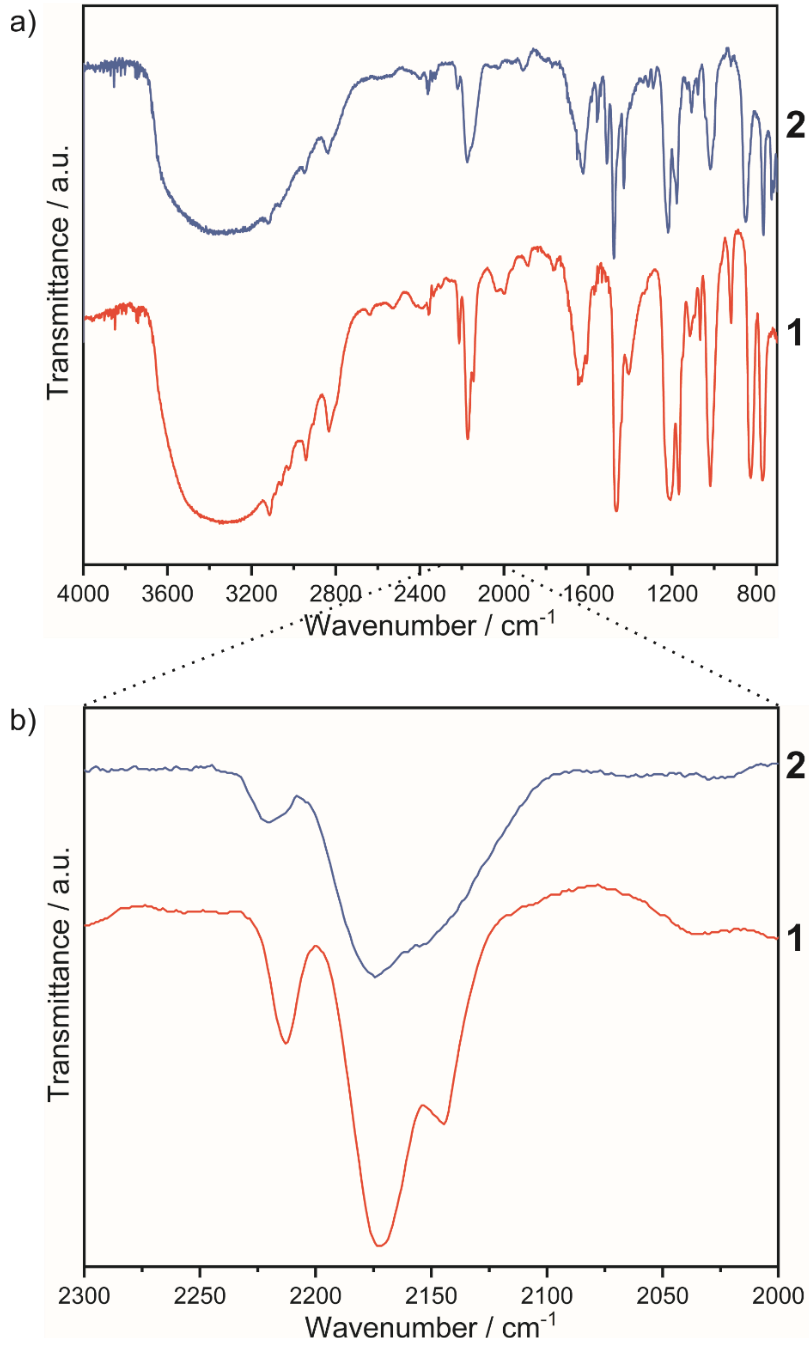

Figure S1. Infrared spectra of **1** and **2** measured at room temperature in the absorption mode for the selected single-crystals in the 4000–700 cm^{-1} range (a) and cyanide stretching vibrations range (b).

Comment: IR spectra of compounds **1** and **2** are very similar. Extensive area of absorption bands in the 1800 – 700 cm^{-1} range is associated with the skeletal $\nu_{C\text{-}H}$, $\nu_{C\text{-}C}$, $\nu_{C\text{-}N}$, and γ vibrations of pyNO and 4-phpyNO ligands in **1** and **2**, respectively. The two N→O streching modes, observed at 1211 and 1168 cm^{-1} (**1**) and 1220 and 1168 cm^{-1} (**2**), are consistent with previous reports on spectroscopic studies on pyNO and 4-phpyNO complexes of transition metal ions.[1,2]. Above 3000 cm^{-1} absorption bands associated with solvent molecules and $\nu_{C\text{-}H}$ ring vibrations are detected. Cyanide stretching vibrations are observed at 2213, 2172 and 2144 cm-1 (**1**) and at 2220, 2174 and 2150 cm^{-1} (**2**), and can be assigned to both bridging (above 2150) and terminal (below 2150) cyanides in $[W^{V}(CN)_8]^{3-}$ building blocks.

a)

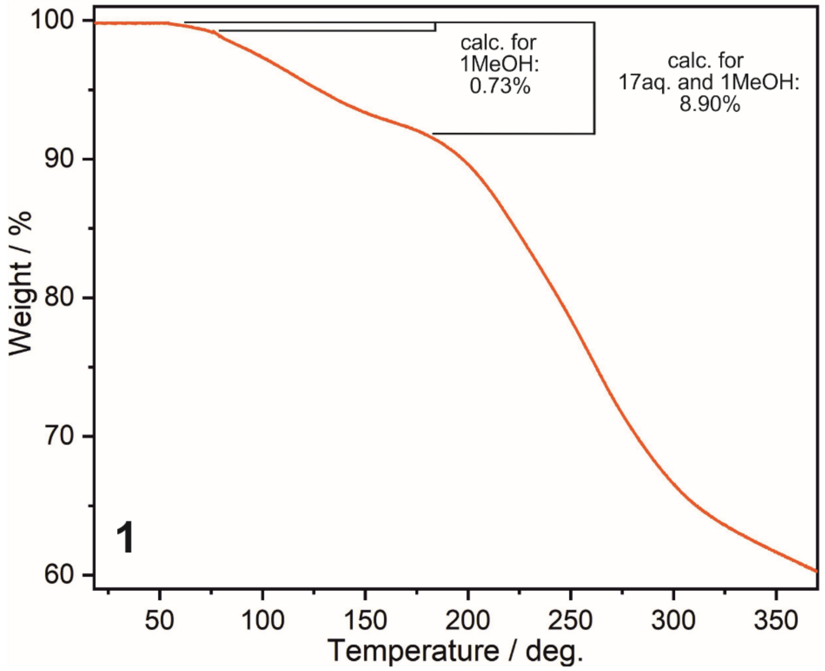

b)

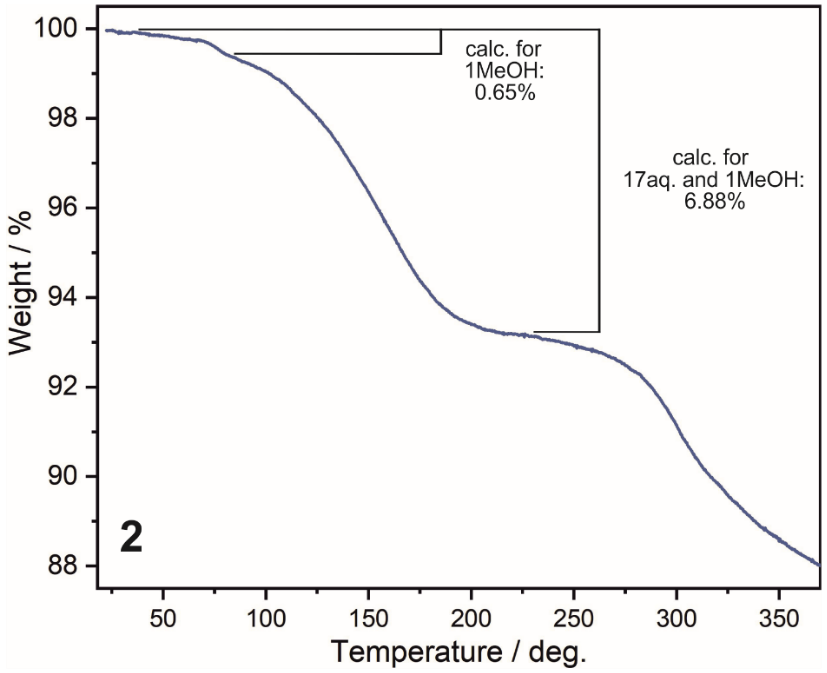

Figure S2. Thermogravimetric curves collected in the temperature range 20 – 375 °C for **1** (a) and **2** (b). The steps related to the loss of solvent molecules are featured.

Comment: Upon heating under air atmosphere the powder samples of **1** and **2** exhibit small decrease of the mass in the range 20 – 90 °C, which is followed by stronger decrease of the mass in the range 100 – 180 °C. The related decrease of the sample mass can be correlated with the amount of solvent molecules determined by CHN analyses (described in the synthetic procedures in main text).

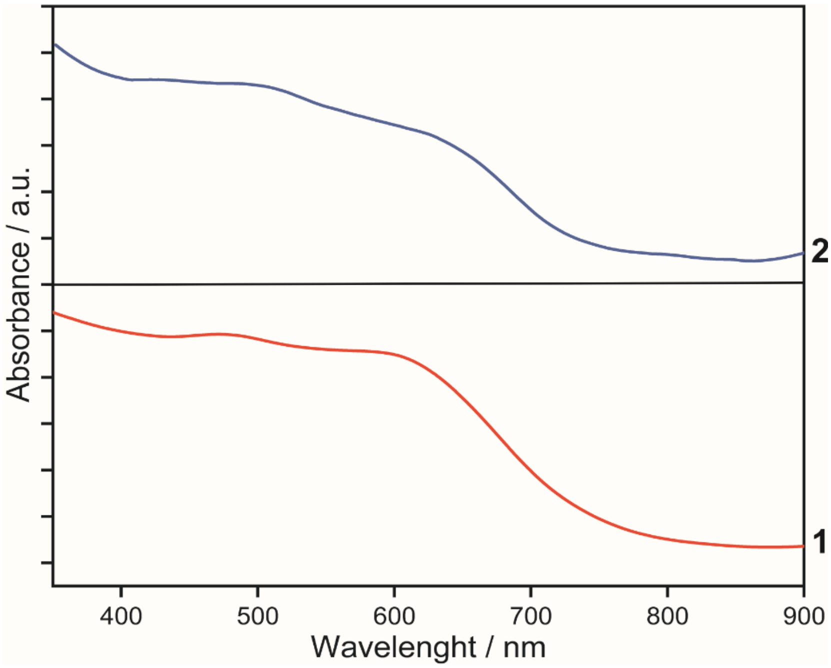

Figure S3. UV–Vis–NIR absorption spectra of **1** and **2** measured at room temperature in the 350 - 900 nm range.

Comment: Both compounds, **1** and **2** exhibit strong absorption in the UV-visible range responsible for the intense dark red colour. Wide range of absorption bands can be explained by the sum of ligand field electronic transitions of $[W^{V}(CN)_8]^{-3}$ ions (UV – 400 cm^{-1} range), d-d electronic transitions of $^{HS}Co^{II}$ ion and metal-to-metal charge transfer (MMCT) electronic transitions. Above spectra are in line with earlier reports.[3,4]

Table S1. Detailed structural parameters of Co^{II} and W^{V} moieties in **1**.

**Parameter**	**1 / Å** (atom labels as in Fig. S4)	**Parameter**	**1 / Å, º** (atom labels as in Fig. S4)
Co1-N1	2.085(10)	W1-C1	2.174(12)
Co2-N3	2.123(16)	W1-C2	2.179(14)
Co2-O1	2.087(15)	W1-C3	2.163(16)
Co3-N2	2.115(12)	W1-C4	2.156(19)
Co3-N5	2.081(12)	W1-C5	2.161(14)
Co3-N7	2.111(15)	W1-C6	2.181(14)
Co3-O2	2.061(12)	W1-C7	2.146(19)
Co3-O3	2.108(11)	W1-C8	2.164(18)
Co3-O4	2.089(12)	W1-CN	174.9(12) to 178.9(13)

Table S2. Detailed structural parameters of Co^{II} and W^{V} moieties in **2**.

**Parameter**	**2 / Å** (atom labels as in Fig. S5)	**Parameter**	**2 / Å, º** (atom labels as in Fig. S5)
Co1-N11	2.066(10)	W1-C11	2.158(14)
Co1-N21	2.081(10)	W1-C12	2.167(13)
Co1-N31	2.096(10)	W1-C13	2.142(13)
Co2-N12	2.113(11)	W1-C14	2.157(13)
Co2-N22	2.090(12)	W1-C15	2.169(13)
Co2-N32	2.130(12)	W1-C16	2.156(15)
Co2-O1M	2.098(9)	W1-C17	2.155(14)
Co2-O2M	2.081(9)	W1-C18	2.174(14)
Co2-O1L	2.066(10)	W2-C21	2.167(12)
Co3-N13	2.121(12)	W2-C22	2.165(12)
Co3-N23	2.083(11)	W2-C23	2.155(13)
Co3-N33	2.079(11)	W2-C24	2.178(14)
Co3-O3M	2.092(10)	W2-C25	2.156(14)
Co3-O4M	2.105(10)	W2-C26	2.172(14)
Co3-O5M	2.087(11)	W2-C27	2.172(16)
Co4-N14	2.079(12)	W2-C28	2.157(16)
Co4-N24	2.100(11)	W3-C31	2.181(14)
Co4-N34	2.080(11)	W3-C32	2.160(14)
Co4-O2L	2.017(10)	W3-C33	2.146(13)
Co4-O3L	2.067(19)	W3-C34	2.158(13)
Co4-O6M	2.119(9)	W3-C35	2.175(13)
Co5-N15	2.109(11)	W3-C36	2.173(16)
Co5-N25	2.116(12)	W3-C37	2.170(14)
Co5-N35	2.119(12)	W3-C38A	2.15(3)
Co5-O4L	2.070(11)	W3-C38B	2.12(3)
Co5-O8M	2.150(9)	W1-CN	175,5(11) to 178,5(13)
Co5-O9M	2.116(11)	W2-CN	174.8(11) to 178,3(15)
		W3-CN	175.5(11) to 178.7(14)

Table S3. Results of Continuous Shape Measure (CSM) analysis for d metal ion complexes in **1**.

Metal complex	CSM parameters			Geometry
	OC-6	TPR-6	-	
$[Co1(\mu\text{-}NC)_6]^{2+}$	0.001	16.655	-	OC-6
$[Co2(\mu\text{-}NC)_3(pyNO)_{1.5}(MeOH)_{1.5}]^{2+}$	0.131	16.304	-	OC-6
$[Co3(\mu\text{-}NC)_3(pyNO)_{1.5}(MeOH)_{1.5}]^{2+}$	0.095	15.538	-	OC-6
	SAPR-8	TDD-8	BTPR-8	
$[W1(\mu\text{-}CN)_5(CN)_3]^{3-}$	2.430	0.520	1.402	TDD-8

Table S4. Results of Continuous Shape Measure (CSM) analysis for d metal ion complexes in **2**.

Metal complex	CSM parameters			Geometry
	OC-6	TPR-6	-	
$[Co1(\mu\text{-}NC)_6]^{2+}$	0.124	16.260	-	OC-6
$[Co2(\mu\text{-}NC)_3(4phpyNO)_1(MeOH)_2]^{2+}$	0.446	13.495	-	OC-6
$[Co3(\mu\text{-}NC)_3(MeOH)_3]^{2+}$	0.084	16.112	-	OC-6
$[Co4(\mu\text{-}NC)_3(4phpyNO)_{1.5}(MeOH)_{1.5}]^{2+}$	0.525/0.460	14.492/13.798	-	OC-6*
$[Co5(\mu\text{-}NC)_3(4phpyNO)_1(MeOH)_2]^{2+}$	0.367	14.150	-	OC-6
	SAPR-8	TDD-8	BTPR-8	
$[W1(\mu\text{-}CN)_5(CN)_3]^{3-}$	1.472	0.725	1.083	TDD-8
$[W2(\mu\text{-}CN)_5(CN)_3]^{3-}$	0.843	1.698	0.980	SAPR-8/BTPR-8
$[W3(\mu\text{-}CN)_5(CN)_3]^{3-}$	1.853/2.936**	1.125/0.658**	0.818/2.401**	TDD-8/BTPR-8**

*Co4 site in compound **2** is coordinated in one axial position by two independent molecules: 4phpyNO ligand or MeOH solvent molecule

W3 site in compound **2 is coordinated in one position by two independent CN^- ligands

Comment: CSM parameter represents the distortion from an ideal geometry. It equals 0 for an ideal polyhedron and increases with the increasing distortion.[5]

Polyhedra codes:

OC-6 – parameter of octahedron geometry related to the O_h symmetry.

TPR-6 – parameter of trigonal prism geometry related to the D_{3h} symmetry.

SAPR-8 – parameter of square antiprism geometry related to the D_{4d} symmetry.

TDD-8 – parameter of triangular dodecaedron geometry related to the D_{2d} symmetry.

BTPR-8 – parameter of bicapped trigonal prism geometry related to the C_{2v} symmetry.

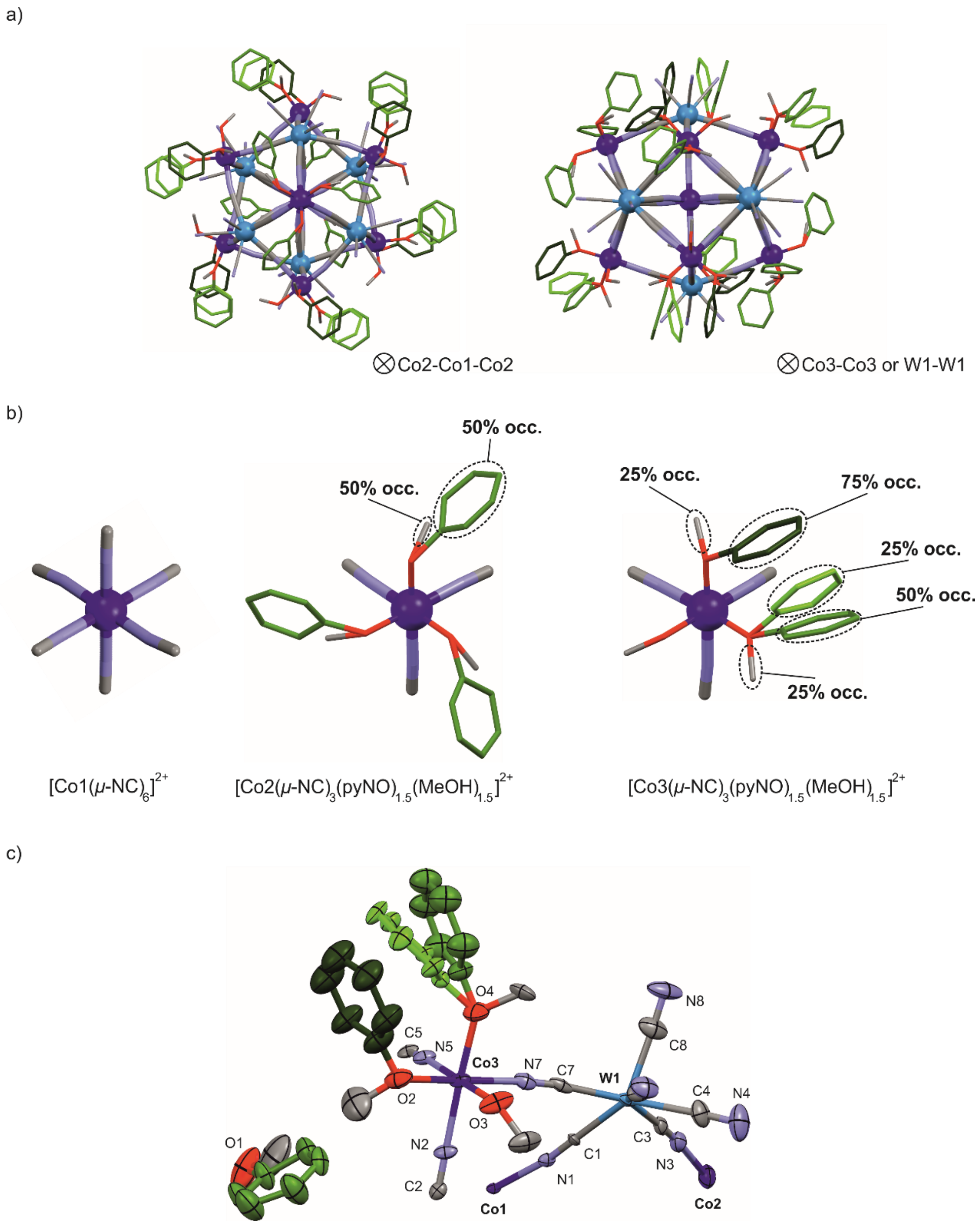

Figure S4. Crystal structure of **1**: a) pentadecanuclear cluster – view along the direction of C2-Co1-Co2 array and Co3-Co3 or W1-W1 array, b) distinction of cationic moieties with the details of individual ligands occupancy and c) asymmetric unit of **1** with labelling scheme for metal ions and their first coordination sphere. Thermal ellipsoids are presented at the 50% probability level. The related bond lengths and angles are collected in Table S1. Hydrogen atoms are omitted for clarity.

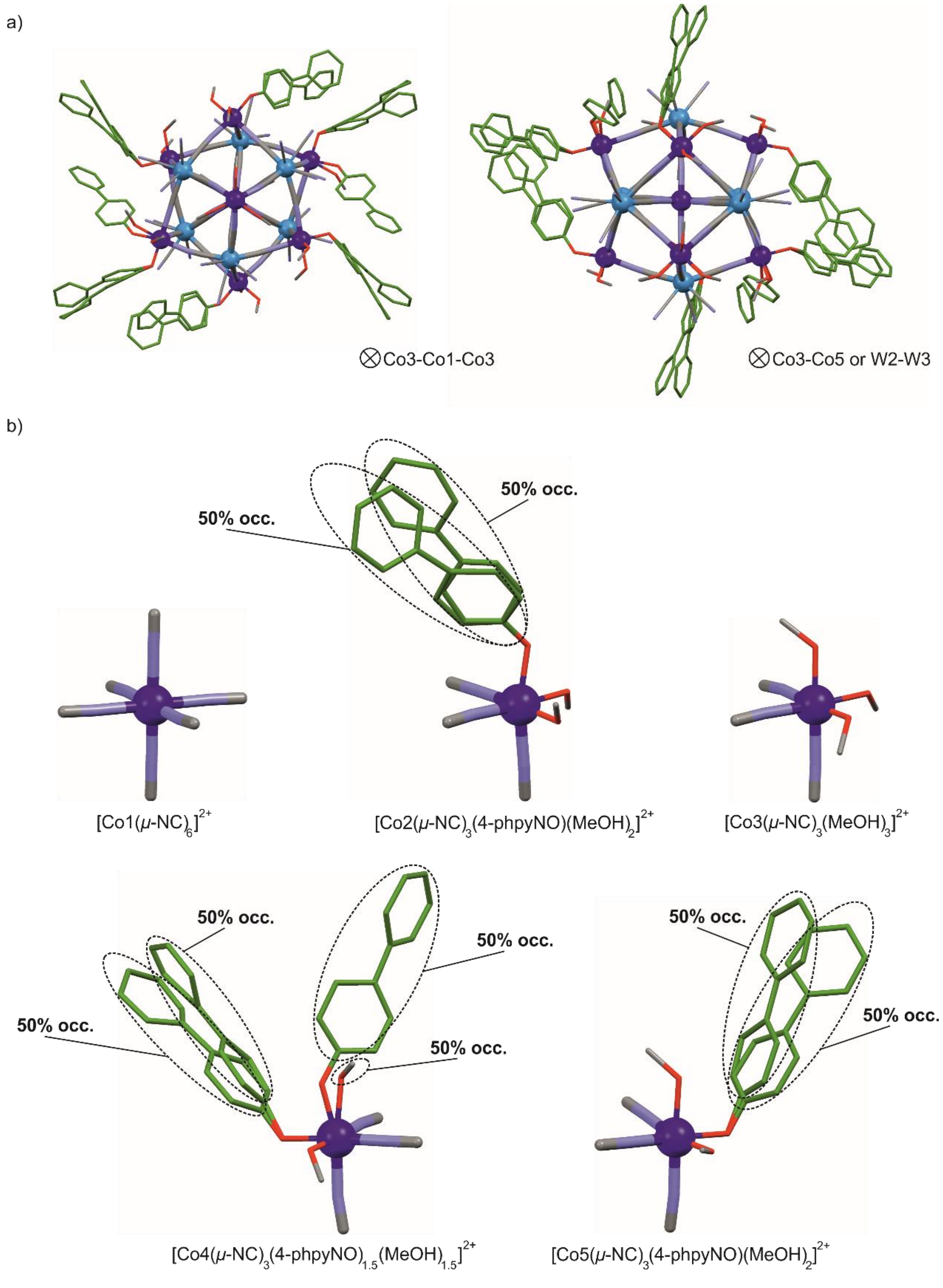

Figure S5. Crystal structure of **2**: a) pentadecanuclear cluster – views along direction of C3-Co1-Co3 array and along the Co3-Co5 or W2-W3 array and b) distinction of cationic moieties with the details of occupancy of individual ligands.

c)

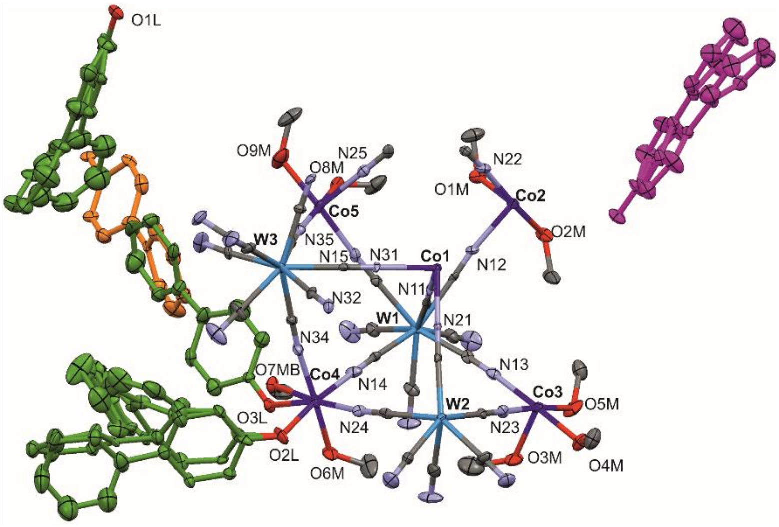

Figure S5 cd. Crystal structure of **2**: c) asymmetric unit of **1** with labelling scheme for metal ions and first coordination sphere of $^{HS}Co^{II}$ units. Thermal ellipsoids are presented at the 50% probability level. The related bond lengths and angles are collected in Table S2. Hydrogen atoms are omitted for clarity.

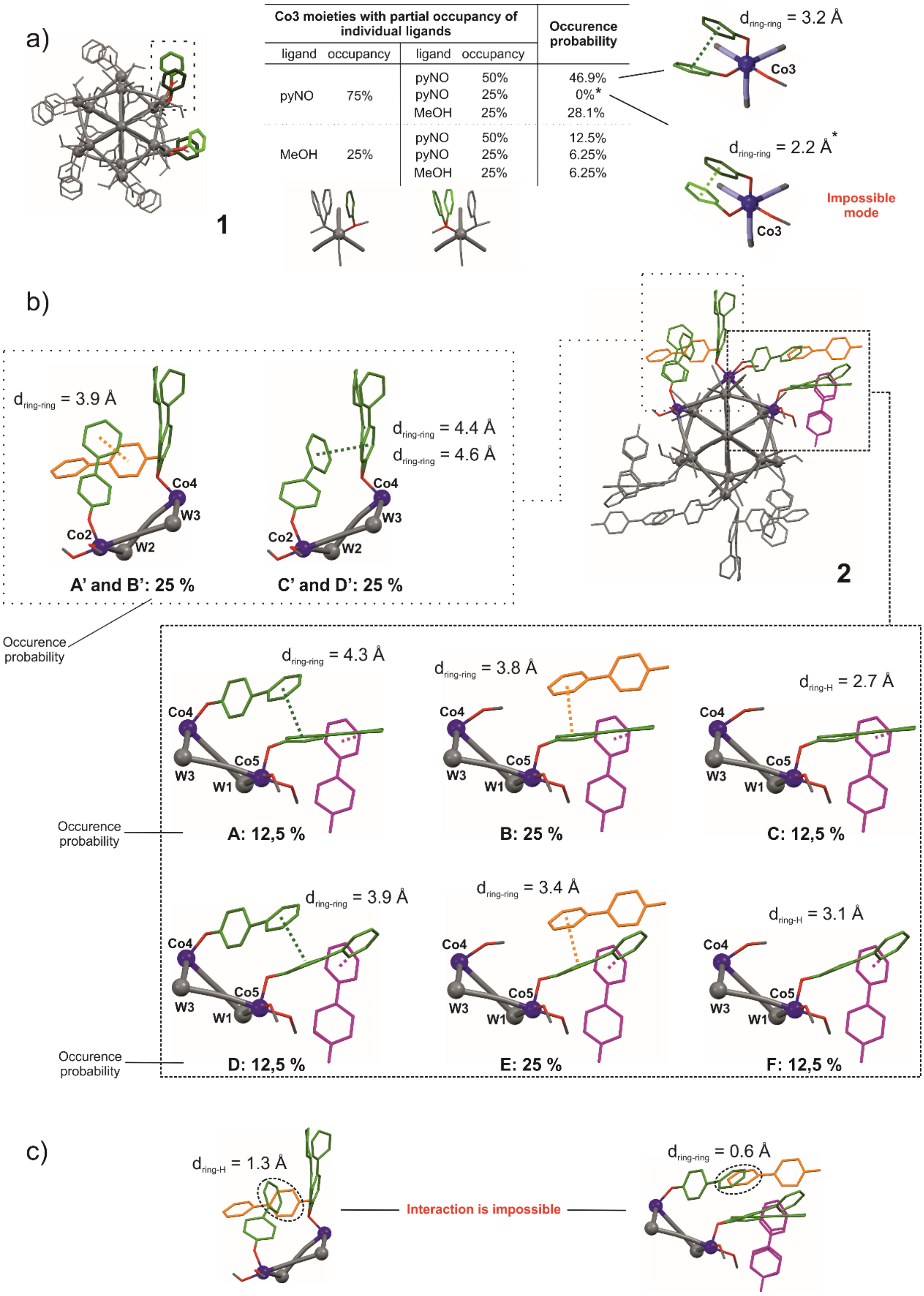

Figure S6. π-π interactions in **1** and **2**: a) face-to-face interactions between pyNO ligands connected to the same Co3 moieties together with an indication of the occurrence probability of each of the coordination environments/ligand arrangement of Co3 moieties in **1**, b) possible types of π-π interactions between 4phpyNO ligands in **2** together with an indication of the occurrence probability of any possible type of interactions in the crystal structure of **2** and c) two examples of situation that preclude the occurrence of this type of ligand arrangement in crystal structure of **2**. Hydrogen atoms are omitted for clarity. Uncoordinated 4-phpyNO ligands are highlighted in magenta and orange.

Comment a): Probability of occurrence of each Co3 coordination environment was determined based on crystallographic occupancy of pyNO ligand (e.g. for two adjacent MeOH: 0.25 * 0.25 = 0.0625); the diagram also indicates a mode impossible to observe in the crystal structure due to the short distance between the position of the related rings atoms (marked with an asterisk).

Comment b) The diagrams show two independent fragments of the structure with π- π interactions: (in the upper part) equally probable four possibilities (**A′**, **B′**, **C′**, **D′**) arrangement of 4-phpyNO ligands – 0.5 at each Co2 and Co4 ions and 0.5 at uncoordinated 4-phpyNO ligand and (in lower part) all six possibilities (A, B, C, D, E and F) for the ligands arrangement at Co4, Co5 and the arrangement of the uncoordinated 4-phpyNO ligand (magenta, occupancy 1.0), including the occurrence (50%) of the uncoordinated (orange) 4-phpyNO ligand (occupancy 0.5).

Comment c) The above occurrence probability were calculated after rejecting all non-physical situations, that cannot occur in a real crystal - two examples are provided at the bottom of **Figure S6**.

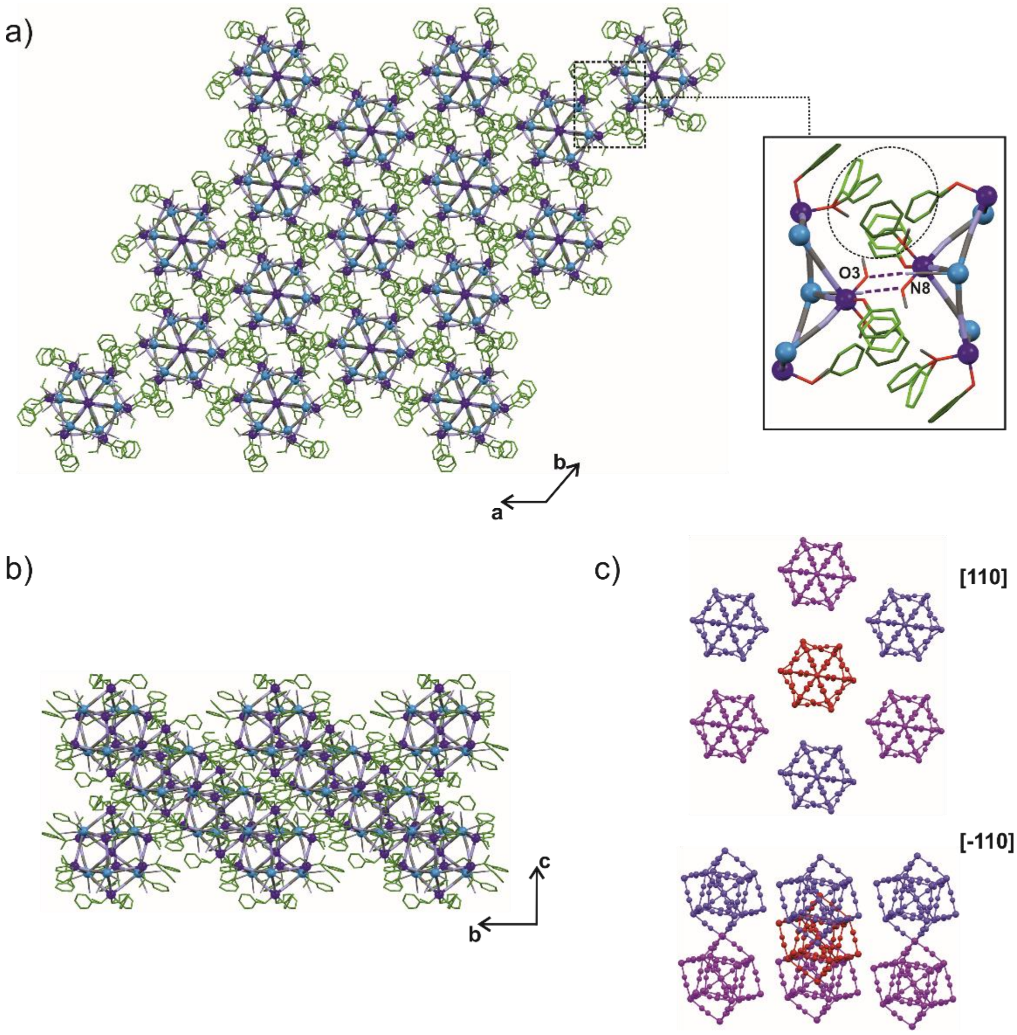

Figure S7. Packing of clusters in **1**: a) view of cluster arrangement along [001] direction with highlighted symmetrical surrounding of clusters pi-pi through stacking and hydrogen-interactions, b) view of cluster arrangement in structure along [100] direction and c) presentation of a symmetrical environment of each cluster. Hydrogen atoms are omitted for clarity.

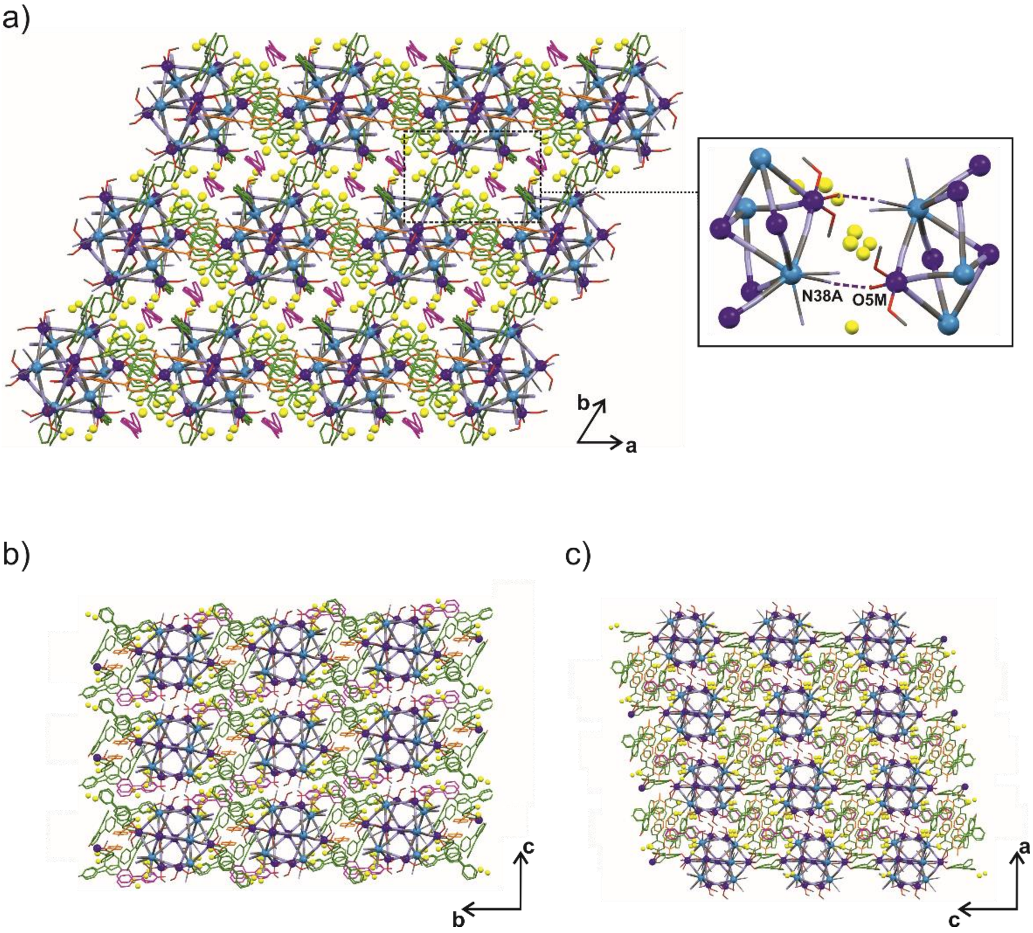

Figure S8. View of cluster arrangement in **2** structure along [001] (a), [100] (b) and [010] (c) directions. Hydrogen bond interactions in the crystal structure of **2** is highlighted. Solvent (MeOH) molecules are marked in yellow and uncoordinated 4-phpyNO ligands are highlighted in magenta and orange. Hydrogen atoms are omitted.

Table S5. Hydrogen bond parameters in **1** and **2**. D – donor atom, A – acceptor atom.

Compound	D	A	D-H / Å	H…A / Å	D-A / Å	D-H…A / Å
**1**	N8	O3	1.896	0.853	2.722	163.09
**2**	N38A	O5M	2.157	0.871	2.918	145.75

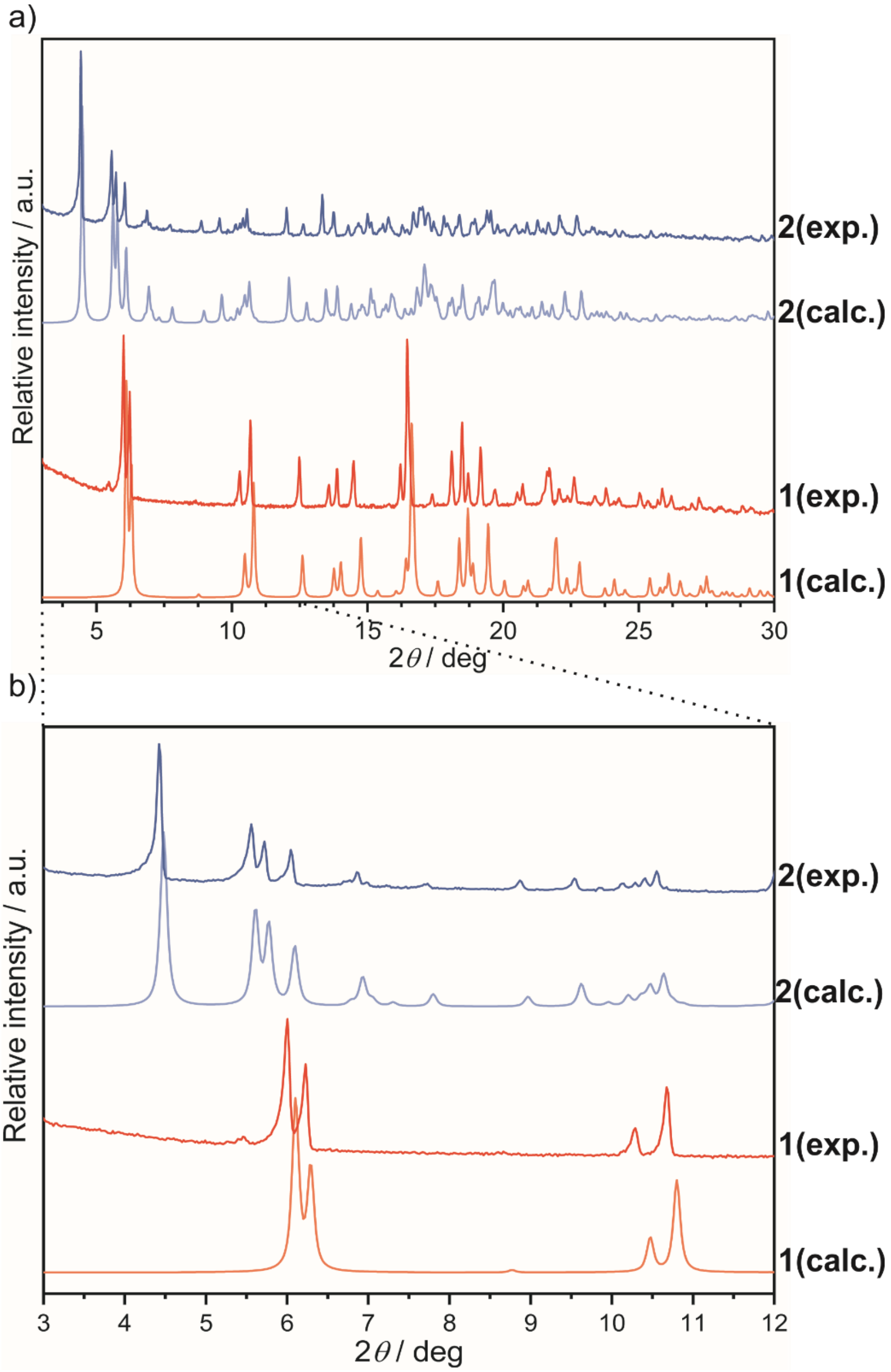

Figure S9. Experimental powder X-ray diffraction (PXRD) pattern of **1** and **2** presented in the broad 2Θ range of 3–40° (a) and in the limited low angle 2Θ range of 3–12° (b). Experimental data were compared with PXRD pattern calculated from the structural model obtain in the single-crystal X-ray diffraction structural analysis.

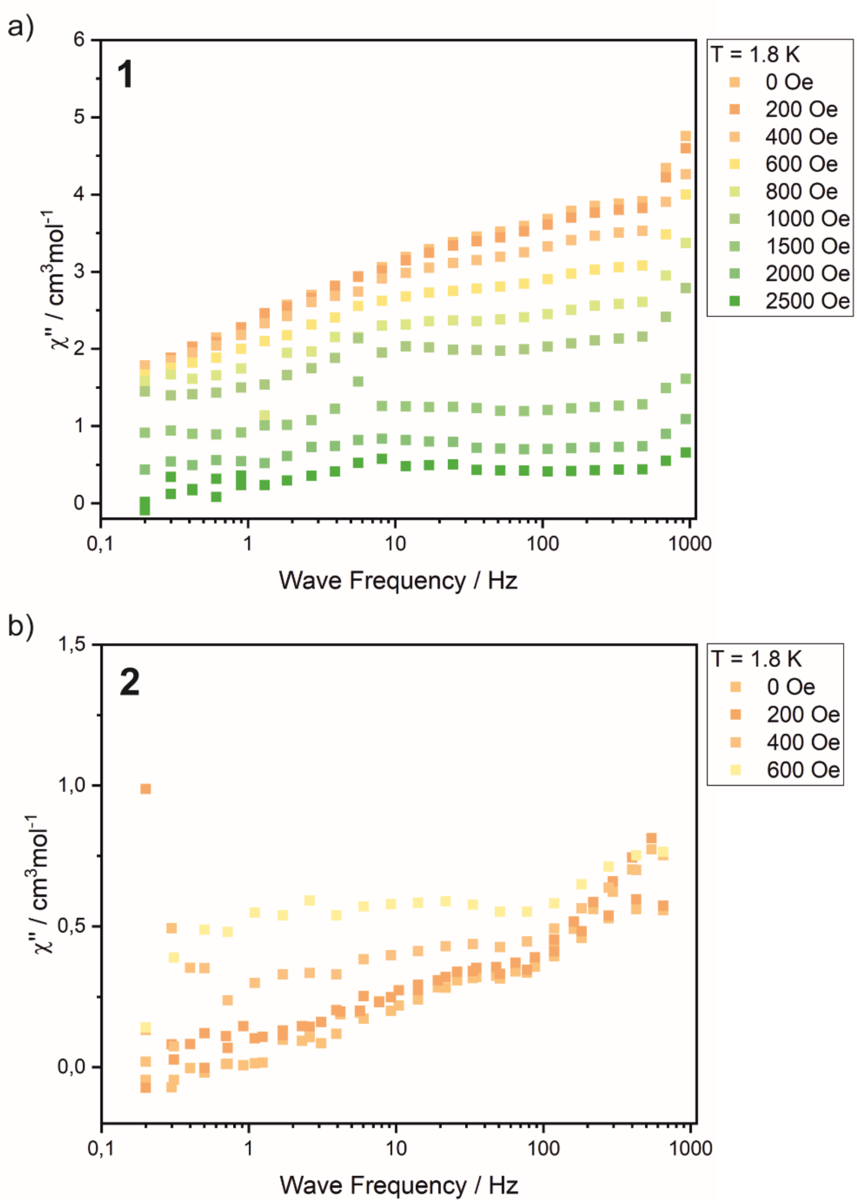

Figure S10. Frequency dependence of out-of-phase magnetic susceptibility, χ'' at 1.8 K in dc field range 0-2500 Oe for **1** (a) and 0-600 Oe for **2** (b).

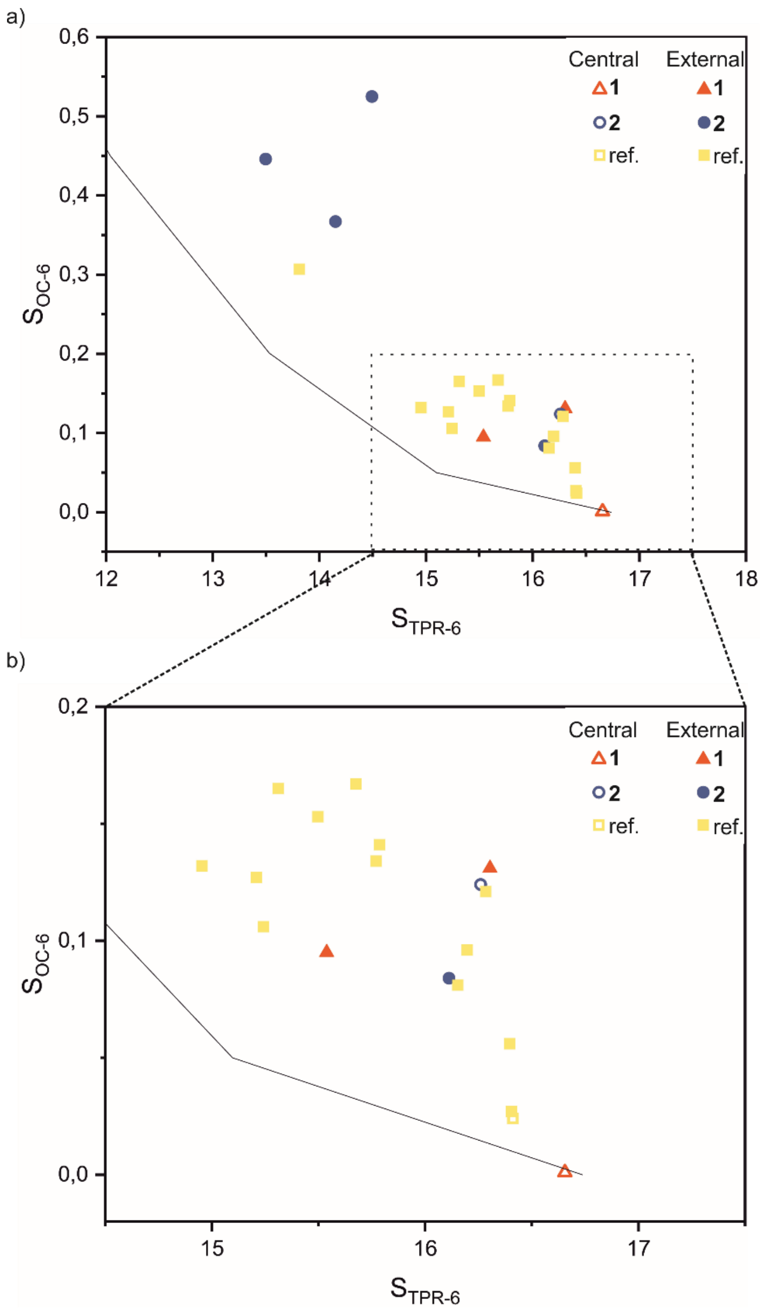

Figure S11. (OC-6)-(TPR-6) shape map with the positions of the coordination polyhedral of Co moieties in **1** (red) and **2** (blue) (a) and the limited shape map range of OC-6 (0.0-0.2) – TPR-6 (14,5-17,5). The same parameters of Co moieties of Co_9W_6 clusters with pyrazine mono-N-oxide (pzmo) and 4,4-bipyridine mono-N-oxide (4,4′-bpmo) are also shown in yellow.[6]

Table S6. The overview of the structural parameters of central Co moieties in cyanido-bridged Co_9W_6 clusters in **1** and **2**.

Compound	Numbering scheme	The adjacent bonds		The opposite bonds		Geometry
**1**	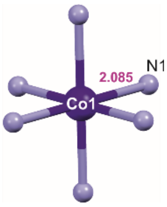	N1-Co1-N1	90.24 / 89.76	N1-Co1-N1	180.00	O_h
**2**	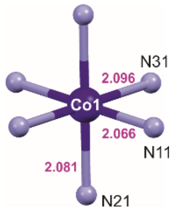	N11-Co1-N21<br>N11-Co1-N3<br>N21-Co1-N31	94.05<br>87.68<br>88.62	N11-Co1-N11<br>N21-Co1-N21<br>N31-Co1-N31	180.00<br>180.00<br>180.00	O_h

Table S7. The overview of the structural parameters of cyanido-bridged Co_9W_6 clusters in **1** and **2**.

Comp.	Structural parameters								Slow relaxation of the magnetization parameters	
	Coordination spheres of the external $^{HS}Co^{II}$	The shortest inter-cluster distance / Å	Co_8 Cube deformations / Å			W_6 octahedron deformations / Å		Number of ligands attached to each Co_9W_6	$\Delta E/k_B$ (K)	τ_0 (s)
			Edges	Diag.	Wall diag.	Edges	Diag.			
1	2 x *cis*-$[N_3O_3]$: $(\mu\text{-NC})_3(L)_{1.5}(MeOH)_{1.5}$<br>6 x *cis*-$[N_3O_3]$: $(\mu\text{-NC})_3(L)_{1.5}(MeOH)_{1.5}$	5.22	7.20 – 7.21 (0.1)	12.47, 12.52	10.18 – 10.20	7.60 – 7.61	10.76	12	–	–
2	2 x *cis*-$[N_3O_3]$: $(\mu\text{-NC})_3(L)_1(MeOH)_2$<br>2 x *cis*-$[N_3O_3]$: $(\mu\text{-NC})_3(MeOH)_3$<br>2 x *cis*-$[N_3O_3]$: $(\mu\text{-NC})_3(L)_{1.5}(MeOH)_{1.5}$<br>2 x *cis*-$[N_3O_3]$: $(\mu\text{-NC})_3(L)_1(MeOH)_2$	7.23	6.74 – 7.65 (0.91)	11.85, 11.89, 12.41, 12.72	10.11 – 10.25	7.24 – 7.93	10.72 – 10.80	7	–	–

References:

1. Mautner, F. A.; Berger, C.; Fischer, R. C.; Massoud, S. S.; Vincente, R. Synthesis, structural characterization and magnetic properties of polymeric azido Mn(II) complexes based on methyl-pyridine-N-oxide co-ligands *Polyhedron* **2017**, 134, 126-134. DOI: 10.1016/j.poly.2017.06.025.

2. Speca, A. N.; Iaconianni, F. J.; Gelfand, L. S.; Pytlewski, L. L.; Mikulski, C. M.; Karaynnis, N. M. Transition metal perchlorate complexes with 4-phenylpyridine N-oxide *J. inorg. Nucl. Chem.* **1978**, 41, 957-961. DOI: 10.1016/0022-1902(79)80070-6.

3. Chorazy S.; Podgajny R.; Nogas, W.; Buda S.; Nitek W.; Mlynarski J.; Rams M.; Koziel M.; Juszynska E.; Vieru V.; Chibotaru L. F.; Sieklucka B. Optical Activity and Dehydration-Driven Switching of Magnetic Properties in Enantiopure Cyanido-Bridged $Co^{II}_3W^V_2$ Trigonal Bipyramids Inorg. Chem. **2015**, 54, 12, 5784-5794. DOI: 10.1021/acs.inorgchem.5b00470.

4. Chorazy, S.; Reczyński, M.; Podgajny, R.; Nogas, W.; Buda, S.; Rams, M.; Nitek, W.; Nowicka, B.; Mlynarski, J.; Ohkoshi, S.; Siekucka, B. Implementation of Chirality into High-Spin Ferromagnetic $Co^{II}_9W^V_6$ and $Ni^{II}_9W^V_6$ Cyanido-Bridged Clusters Cryst. Growth Des. **2015**, 15, 3573–3581. DOI: 10.1021/acs.cgd.5b00321.

5. L lunell, M.; Casanova, D.; Cirera, J.; Bofill, J.; Alemany, P.; Alvarez, S.; Pinsky, M.; Avnir, D. SHAPE v. 2.1. Program for the Calculation of Continuous Shape Measures of Polygonal and Polyhedral Molecular Fragments, University of Barcelona: Barcelona, Spain, **2013**.

6. Kobylarczyk, J.; Augustyniak, K.; Chorazy, S.; Nowicka, B.; Pinkowicz, D.; Kozieł, M.; Muziol, T.; Podgajny, R. Cyanido-Bridged Clusters with Remote N-Oxide Groups for Branched Multimetallic Systems *Cryst. Growth Des.* **2018**, 18, 4766–4776. DOI: 10.1021/acs.cgd.8b00860.
